# The CAST study protocol: a cluster randomized trial assessing the effect of circumferential casting versus plaster splinting on fracture redisplacement in reduced distal radius fractures in adults

**DOI:** 10.1186/s12891-021-04238-0

**Published:** 2021-04-20

**Authors:** Britt Barvelink, Max Reijman, Niels W. L. Schep, Vanessa Brown, Gerald A. Kraan, Taco Gosens, Suzanne Polinder, Erwin Ista, Jan A. N. Verhaar, Joost W. Colaris, on behalf of the CAST study group, Ninka Slebioda, Mathieu M. E. Wijffels, Marna G. Bouwhuis, Flip van Beek, Daphne A. van Rijssel, Mark R. de Vries, Michiel Leijnen, Peer van der Zwaal, Merel van Loon, Alexander P. A. Greeven, Lenneke Scholtens, Ruud L. M. Deijkers, Marike C. Kokke, Milko M. M. Bruijninckx

**Affiliations:** 1 Department of Orthopedic Surgery, Erasmus MC, University Medical Center Rotterdam, P.O. Box 2040, 3000 Rotterdam, CA The Netherlands; 2 Department of Trauma Surgery, Maasstad Hospital, Rotterdam, The Netherlands; 3 Department of Emergency Medicine, Franciscus Hospital, Rotterdam, The Netherlands; 4 Department of Orthopedic Surgery, Reinier de Graaf Gasthuis, Delft, The Netherlands; 5 Department of Orthopedic Surgery, Elisabeth-Tweesteden Hospital, Tilburg, The Netherlands; 6 Department of Public Health, Erasmus MC, University Medical Center Rotterdam, Rotterdam, The Netherlands; 7 Department of Internal Medicine – Nursing Science, Erasmus MC, University Medical Center Rotterdam, Rotterdam, The Netherlands

**Keywords:** Fracture, Bone, Distal radius fracture, Cast, Splint, Fracture displacement, Cost-effectiveness

## Abstract

**Background:**

There is no consensus concerning the optimal casting technique for displaced distal radius fractures (DRFs) following closed reduction. This study evaluates whether a splint or a circumferential cast is most optimal to prevent fracture redisplacement in adult patients with a reduced DRF. Additionally, the cost-effectiveness of both cast types will be calculated.

**Methods/design:**

This multicenter cluster randomized controlled trial will compare initial immobilization with a circumferential below-elbow cast versus a below-elbow plaster splint in reduced DRFs. Randomization will take place on hospital-level (cluster, *n* = 10) with a cross-over point halfway the inclusion of the needed number of patients per hospital. Inclusion criteria comprise adult patients (≥ 18 years) with a primary displaced DRF which is treated conservatively after closed reduction. Multiple trauma patients (Injury Severity Score ≥ 16), concomitant ulnar fractures (except styloid process fractures) and patients with concomitant injury on the ipsilateral arm or inability to complete study forms will be excluded. Primary study outcome is fracture redisplacement of the initial reduced DRF. Secondary outcomes are patient-reported outcomes assessed with the Disability Arm Shoulder Hand score (DASH) and Patient-Rated Wrist Evaluation score (PRWE), comfort of the cast, quality of life assessed with the EQ-5D-5L questionnaire, analgesics use, cost-effectiveness and (serious) adverse events occurence. In total, 560 patients will be included and followed for 1 year. The estimated time required for inclusion will be 18 months.

**Discussion:**

The CAST study will provide evidence whether the type of cast immobilization is of influence on fracture redisplacement in distal radius fractures. Extensive follow-up during one year concerning radiographic, functional and patient reported outcomes will give a broad view on DRF recovery.

**Trial registration:**

Registered in the Dutch Trial Registry on January 14th 2020. Registration number: NL8311.

## Background

Displaced distal radius fractures (DRF’s) are very common in the adult population and their incidence is still increasing because of the ageing population [1, 2]. In the Netherlands, the incidence of DRFs is estimated at 20 per 10.000 persons per year [3]. Two-thirds of DRFs in adults are displaced and require closed reduction [4]. After successful reduction, DRFs are generally immobilized using a non-circular splint or a circumferential cast. Unfortunately, a large number of reduced DRF’s (32–64%) redisplace during cast immobilization in the first treatment weeks [5–7]. Whereas redisplacement of DRFs was previously accepted or reduced in a second attempt, nowadays these redisplaced fractures are generally treated surgically [8, 9].

Although surgical reduction and fixation generally results in a satisfying outcome, preventing fracture redisplacement and thereby preventing surgery would be the preferred scenario. Therefore, it is important to discover factors that could predict or prevent fracture redisplacement. Studies focusing on this topic are mostly assessing non-modifiable factors predicting fracture redisplacement [5–7, 10]. Known factors enhancing the risk on fracture displacement are female gender, age > 60 years and fractures with dorsal comminution [10]. However, it is unknown if type of cast immobilization is of influence.

Existing literature concerning the role of cast immobilization on fracture re-displacement is scarce and inconclusive [11]. Recently, Caruso et al. (2019) performed a randomized controlled trial comparing the effect of above-elbow casting and below-elbow casting on maintaining reduction and found similar results in both groups [12]. Patient-reported outcome after 1-year follow-up did not differ amongst both treatment groups. A randomized controlled trial by Wik et al. (2009) evaluated fracture alignment during 5 weeks follow-up in 72 patients with displaced DRF’s treated with dorsal splinting versus circumferential casting [13]. The circumferential casting group showed a significantly better result for radial length at 5 weeks but no difference concerning dorsal angulation. Recently, our research group conducted a retrospective study in 500 patients with reduced DRFs and found significantly less fracture redisplacement in fractures treated with circumferential casting compared with splinting, namely 17% versus 29% [14].

The choice for a splint or circumferential cast is often based on the treating physician’s preference [11]. A splint could be considered easy and quick to apply which is favorable at busy emergency departments, but it can loosen easily. One argument for not applying a circumferential cast initially after reduction is the risk of pain and comprised circulation due to swelling. Several studies however found none or mild differences in pain complaints comparing circumferential casting with splinting [13, 15, 16].

This study aims to clarify if type of cast immobilization influences maintaining fracture alignment in reduced adult distal radius fractures. A cluster randomized controlled trial is designed to compare the treatment of reduced DRFs with a splint or a circumferential cast. Radiological, functional and patient-reported outcomes are studied during a one-year study period.

## Methods/design

This manuscript is written according to the Consolidated Standards for Reporting Trials (CONSORT statement) and Standard Protocol Items: Recommendations for Interventional Trials (SPIRIT guidelines) [17, 18].

### Objectives

The primary objective is to assess which type of cast, a splint versus a circumferential cast, is most optimal to prevent fracture redisplacement in adult patients with a reduced DRF.

As secondary objectives, we assess which type of casting results in fewer surgical interventions and complications. Thereby we will assess cost-effectiveness, the comfort of the cast, pain scores, functional outcome, patient-reported outcome and quality of life.

### Design, participants, interventions and outcomes

#### Study design and randomization

For this study, a multicenter cluster randomized design is used. All participating hospitals are located in the Netherlands and include the following 10 centres; Alrijne hospital (Leiderdorp), Elisabeth-Tweesteden hospital (Tilburg), Erasmus MC University Medical Center (Rotterdam), Franciscus hospital, location Gasthuis and Vlietland (Rotterdam and Schiedam), Haga Teaching hospital (Den Haag), Haaglanden Medical Center (Den Haag), IJsselland hospital (Capelle aan den IJssel), Maasstad hospital (Rotterdam), Reinier de Graaf Gasthuis (Delft) and St. Antonius Hospital (Utrecht and Nieuwegein).

Two types of cast immobilization will be compared in this trial. Randomization at patient level will be challenging because of the 24/7 availability of the Emergency Department (ED), leading to a high number of treating physicians. To overcome the potential of many protocol violations, we chose to randomise on hospital-level with a cross-over point halfway the needed inclusions per hospital (i.e. after 31 inclusions). This means all patients in one hospital will receive the same intervention, which will change after half of the number of patients are included. This cross-over design is used to overcome potential non-eligibility of both groups because patient populations can differ amongst hospitals. Secondly, possible bias due to existing experience with one of the techniques in each hospital will be diminished. Before the start of the study, an independent researcher not involved in the study, randomly allocated the starting treatments amongst the participating hospitals.

#### Study population

##### Participants and recruitment

The study population will consist of adult patients who visit the ED of participating hospitals with a distal radius fracture needing reduction. Fractures who should be reduced conform to the Dutch guideline meet one or more of the following criteria: > 15° of dorsal angulation, > 20° of volar angulation, < 15° of radial inclination, > 3 mm of radial shortening and > 2 mm intra-articular step-off or gap.

Patients that match the inclusion criteria are informed about the study by the treating physician before closed reduction. All patients receive written information, namely the patient information folder (PIF) and a short information folder containing the study aim, contact information of the local hospital and general information about cast immobilization. Because of the acute status of a sustained fracture, patients will be asked for participation in the study directly after diagnosis of a displaced DRF. Written informed consent is obtained before the study procedure starts.

##### Inclusion criteria

- Age ≥ 18 years
- Distal radius fracture requiring closed reduction

##### Exclusion criteria

- Concomitant ulnar fracture (styloid process fracture not encountered)
- Multiple trauma patients (Injury Severity Score (ISS) ≥ 16)
- Concomitant injuries to the ipsilateral extremity, interfering with the treatment of the DRF
- Inability to complete study forms due to any mental status or insufficient understanding of the Dutch language

#### Study procedures and timeline

Measurements will take place at 7 time points as shown in Table 1. These time points are: baseline (T0), 1 week (T1), 2 weeks (T2), 5 weeks (T3), 3 months (T4), 6 months (T5) and 1 year (T6) after inclusion in the study. At T0, baseline characteristics will be gathered. An inclusion form will be filled in by the treating physician providing fracture and treatment-specific information. Second, patients receive a questionnaire, providing predominantly patient and injury-specific information. The list of baseline characteristics is shown in Table 2. Patients receive questionnaires at T1 to T6. These questionnaires are carried out by email with the use of data capture system GemsTracker [19]. GemsTracker (GEneric Medical Survey Tracker) is a secure web-based application for distribution of questionnaires and forms during clinical research and quality registrations. Receiving questionnaires on paper is optional, as well as telephone interviews. Reminders will be automatically sent. When the patient does not respond to emails, we will contact the patient by telephone. Posterior-anterior (PA) and lateral radiographs of the wrist will be taken at T0 (before and after reduction) and during follow-up at T1 –T3. Physical examination of the wrist will take place at T4 and will be performed by the researcher. If patients are not able to visit the hospital, a home visit will be offered to improve the follow-up.

**Table 1:** Overview of measurements

	T0	T1	T2	T3	T4	T5	T6
	Baseline	1 week	2 weeks	5 weeks	3 months	6 months	1 year
Inclusion form	x						
X-rays^a^	x	x	x	x			
Function tests ^b^					x		
*NRS*		x	x	x	x	x	x
*Comfort of cast*		x	x				
*Analgesic use*		x	x	x	x	x	x
*EQ 5D5L*		x^c^		x	x	x	x
*QDASH*				x	x	x	x
*PRWHE*				x	x	x	x
*MCQ-iMTA*				x	x	x	x
*PCQ-iMTA*				x	x	x	x

^a^ X-rays before and one after reduction

^b^ Concerning ROM, grip strength and specific tests

^c^ EQ 5D5L status pre-fracture and post-fracture sustainment

Questionnaires are written in *italics*

**Table 2:** Baseline characteristics

Inclusion form	Questionnaire
Date of emergency room visit	Sex
Affected wrist	Date of birth
Method of reduction	Length
Reduction executed by: *(*e.g. *specialist, resident, intern, nurse, cast technician)*	Weight
	Hand dominance
Number of reduction attempts	Mechanism of injury
Type of cast applied	Smoking status
Application of cast executed by: *(*e.g. *specialist, resident, intern, nurse, cast technician)*	General medical history
	Previous injuries of the affected extremity
Neurovascular status of fractured hand/wrist	

#### Interventions

**Fig. 1:**
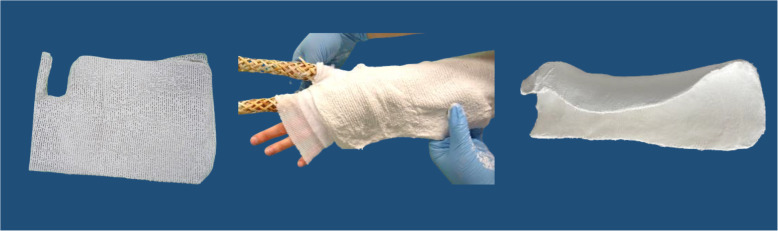
Plaster splint

**Fig. 2:**
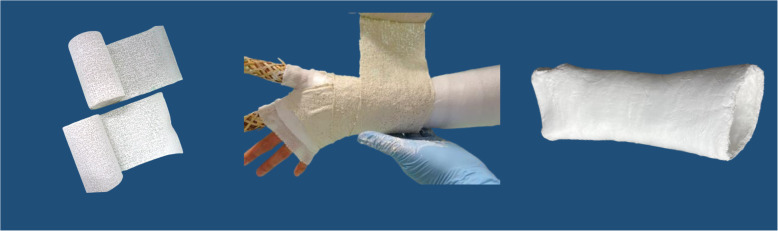
Circumferential cast

We will compare two casting options that are applied directly after reduction: a plaster of Paris envelope splint (further called splint) versus a below-elbow forearm cast (further called circumferential cast). Both interventions are shown in Figs. 1 and 2. The splint or circumferential cast will be applied by an ED nurse, a cast technician, the physician or a physician assistant. Both interventions will be implemented by education and training. Education will be available at the ED’s at all times by means of an instruction video and an instruction poster. The need for training of both casting techniques is evaluated before the start of the study and differs amongst participating hospitals. We chose hospital individualized training instead of a general training program because participating hospitals differ in: the currently used treatment, the experience of ED nurses with both casting techniques and the availability of cast technicians at the ED. In case extra training is needed, training is organized by local cast technicians, in accordance with the research group.

#### Outcome

##### Primary outcome

The primary outcome of this study is the occurrence of fracture re-displacement of the initial reduced DRF assessed on PA and lateral radiographs at 1, 2 and 5 weeks after reduction. Displacement of the radius is defined by the Dutch guideline: > 15° of dorsal angulation, > 20° of volar angulation, < 15° of radial inclination, > 3 mm of radial shortening and > 2 mm intra-articular step-off or gap. Measurements will be digitally carried out in the Picture Archiving and Communication System (PACS) on standard PA and lateral radiographs of the wrist. PA and lateral radiographs will be taken conform standardised procedures. An Intraclass Correlation Coefficient (ICC) will be calculated to assess inter- and intra-observer variability in radiograph measurements. For this, 50 radiographs will be measured twice by BB and NS.

##### Secondary outcomes

An overview of measurements is shown in Table 1.

- Comfort of the cast assessed using a self-developed questionnaire at T1 and T2.
- Severity of pain evaluated with the Numeric Rating Scale (NRS) at T1 to T6.
- Patient-reported recovery of function assessed using the Quick Disabilities of the Arm, Shoulder and Hand questionnaire (Q-DASH) and the Patients-Rated Wrist Hand Evaluation questionnaire (PRWHE). Both questionnaires are validated for assessing functional outcome in patients with a DRF [20–22]. Questionnaires will be carried out at T3 to T6.
- Quality of life will be assessed using the 5-level EuroQol (EQ-5D-5L) [23] scoring questionnaire at T1 (pre- and postfracture state) and T3 to T6.
- Cost-effectiveness will be measured using the Medical Consumption Questionnaire (iMCQ) and the Production Consumption Questionnaire (iPCQ) [24]. Questionnaires will be sent at T3 to T6.
- Recovery of function will be evaluated by physical examination at T4.
  - Range of motion (ROM) of the wrist will be measured with a goniometer. ROM concerns dorsal flexion, volar flexion, radial deviation, ulnar deviation, pronation and supination.
  - Grip strength will be measured with a Jamar hydraulic hand dynamometer. Patients get three attempts on both sides. The maximum score for each side is used in the analyses.
  - Specific testing of the wrist and hand will be performed namely:
    - distal radioulnar joint (DRUJ) stability tested with the DRUJ ballottement test [25]
    - opposition of the thumb using the Kapandji score [26]
    - finger stiffness will be measured with finger-to-palm distance, tested from the tip of the finger to the distal palmar crease when the fingers are in maximal active flexion [27]
  ROM and grip strength on the injured side will be compared with the uninjured side.
- Number of conversions to surgical treatment and other (serious) adverse events will be monitored and are listed at 1.9.

#### Modified follow-up by partial exclusion

**Fig. 3:**
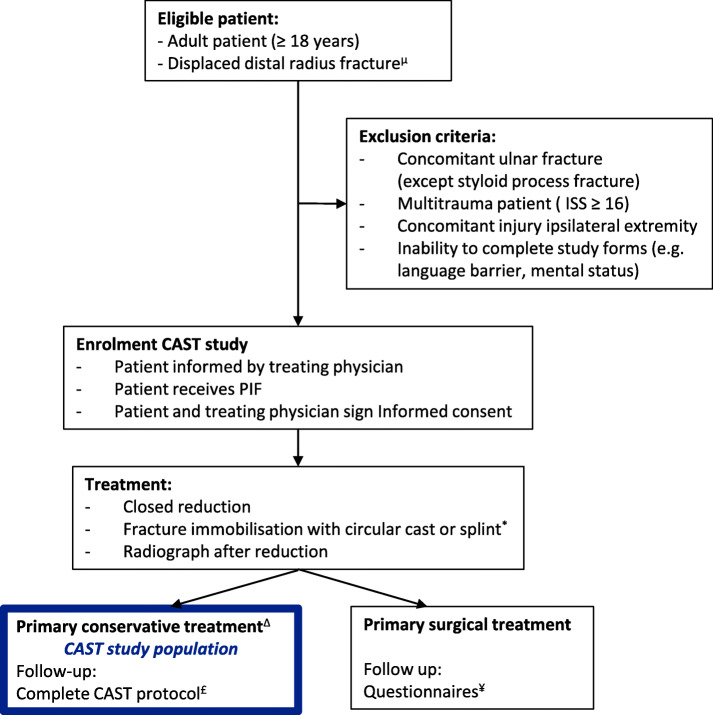
CAST study follow-up flowchart. ^*^Type of treatment depends on randomization status of the hospital. ^*μ*^ Unacceptable alignment conform the Dutch guideline: > 15° of dorsal angulation, > 20° of volar angulation, < 15° of radial inclination, > 3 mm of radial shortening and > 2 mm intra-articular step-off or gap. ^∆^ Cast immobilization, at least until the first control radiographs are taken. ^£^ All measurements encountered in Table 1. ^¥^ All questionnaires encountered in Table 1

Patients are recruited before fracture reduction because immobilization takes place immediately after reduction. A part of included patients will receive surgical fixation in the first treatment week, due to unsuccessful fracture reduction. Consequently, it is unavoidable to include patients who will eventually be unsuitable to answer the primary research question. Patients receiving surgical fixation before the first follow-up appointment (T1) will not be encountered in the needed patient numbers. Recovery of these patients will be evaluated by questionnaires only (Fig. 3).

#### (serious) adverse events reporting

All adverse events reported by the patient or observed by the treating physician or researcher will be recorded. Serious adverse events (SAE) will be reported through the web portal *ToetsingOnline* of the Central Committee on Research Involving Human Research (Dutch CCMO) to the Medical Research Ethics Committee of Erasmus Medical Center in Rotterdam, which approved the protocol. SAE reporting will take place within 7 days after the sponsor has first knowledge of a serious adverse event resulting in death or is life-threatening. All other SAE will be reported within 15 days.

Adverse events are defined as:

- Cast or splint related problems
- Fracture redisplacement treated with surgical reduction and fixation
- Complex regional pain syndrome (CRPS) defined conform the Budapest criteria
- Disabling fracture mal-union or non-union

Study related serious adverse events are defined as:

- Compartment syndrome

#### Sample size

The sample size calculation is based on the results of our recent retrospective study [14]. We hypothesize that fracture redisplacement occurs in 10% of circumferential casted patients versus 20% in splinted patients. A total of 500 patients is needed to detect superiority of a circumferential cast. The sample size calculation is based on a mixed-effects logistic regression, to account for clustering using a random intercept for the hospitals. The intra-class correlation coefficient between the different hospitals for the proportion of fracture redisplacement is assumed to be 0.06, which is generally reported in the literature for hospital processes. From the expected proportions of redisplacement in the two groups, we calculated the hospital-specific log odds of secondary displacement in the circular cast group, equal to − 2.275 and log odds ratio of redisplacement between the two groups equal to 0.84. This calculation is based on the formula that links the cluster-specific coefficients in the mixed-effects logistic regression with the population coefficients averaged over the hospitals. With a power of 93%, 50 patients per hospital need to be included, resulting in a total number needed of 500 (10 participating hospitals). Additionally, we also calculated the needed number of patients using a two-sample test for proportions. Using the same assumptions, namely difference between groups, a significance level of 0.05 and a power of 90%. This resulted in 490 needed patients in total. Accounting for a 10% loss to follow-up, a total of 560 (280 patients per group) are required.

To improve awareness of the study in all participating centers and thereby advert for reaching targeted sample size, a local investigator is appointed in each hospital. This local investigator is a physician who supervises the study. This person is easily accessible and will promote the study on a regular base. We send a newsletter every 2 months to inform about the study progression.

### Data analysis

General descriptive statistics will be performed on baseline patient and fracture characteristics. Patients will be analyzed according to the intention-to-treat principle.

#### Primary outcome

The primary outcome, fracture re-displacement, will be analyzed using a mixed-effects logistic regression. To account for clustering, a random intercept for the hospitals will be used. Fixed effects will be the covariates we adjust for as reported in the literature, namely age, presence of osteoporosis, and fracture characteristics. If new prognostic factors will be identified and reported in the literature, these factors will also be added as covariates.

#### Secondary outcomes

For secondary outcomes, trends between baseline and follow-up time points (T0-T6) will be assessed using linear mixed models for repeated measures. This accounts for comfort of the cast, recovery of function and grip strength, pain severity (NRS), Q-DASH scores, PRWHE and EQ-5D-5L scores. The number of conversions to surgical fixation and complications will be determined using Fisher Exact or Chi-square test, depending on the magnitude of results.

#### Cost-effectiveness analysis

Both cost-effectiveness (CEA) and cost-utility (CUA) analysis will be performed from a societal perspective. For the calculation of medical costs, we will use charges as published in Dutch guidelines as a proxy of real costs. The unit per price of the cast and splint application in patients with DRF will be calculated with the micro-costing method. Intramural costs (i.a. additional diagnostics, number of hospital visits, in case of hospital admission the length of stay etc.) are collected from the electronic health record. Productivity costs will be registered in detail by the iPCQ. The iMCQ and the iPCQ are validated by the Institute of Medical Technology Assessment (Erasmus University, Rotterdam, The Netherlands).

The difference in costs and effects of a circumferential cast instead of a splint will be calculated as incremental cost-effectiveness ratio (ICER). The primary effect outcome measures will be the number of re-displacements for the CEA and quality-adjusted life years (QALY) for the CUA. QALYs will be measured, based on the Dutch tariff for the EQ-5D-5L.

The sensitivity analysis will assess the robustness of the results to changes in costs and effect parameters. Bootstrapping with 5000 replications will be used to estimate 95% confidence intervals around cost differences and the uncertainty surrounding the ICERs. This will be graphically presented on cost-effectiveness planes and acceptability curves using the net benefit framework [28, 29]. For the time horizon of 1 year, discounting is not necessary.

### Data management

All data are handled confidentially and anonymized in compliance with the Dutch Personal Data Protection Act. Personal data of participants will be changed by a study number. This number is used for all study documentation, study reports and publications. The key of this study number will be handled by an independent researcher. During the study period, all data will be collected and managed using GemsTracker electronic data capture tools hosted at Erasmus Medical Center [19]. Paper case report forms are entered in GemsTracker by the researcher and the original paper case forms will be filed in the investigator site file at the recruiting hospital. All data is stored for 15 years.

#### Data monitoring

Since the study is labelled as low risk, a data safety monitoring board is not required. However, the study will be monitored at least once a year by an independent monitoring board. A written report will be available from all monitors.

The investigator will submit a progress report to the accredited Medical Research Ethics Committee of Erasmus Medical Center in Rotterdam throughout the clinical trial annually. This will consist of the date of inclusion of the first subject, numbers of subjects included and numbers of subjects that have completed the trial, SAEs, other problems and amendments.

#### Dissemination

We plan to present the study results at (inter) national conferences and submit the manuscript to general peer-review journals. We aim to implement the study results in the Dutch guideline for DRFs.

## Discussion

This study is an open-label trial. Allocated treatments are visually different for the treating physician and the patient. Randomization status will therefore not be blinded.

Heterogeneity of the study population concerning fracture characteristics and age could be pointed out as a limitation. However, this pragmatic study tries to represent the actual patient population.

## Data Availability

The datasets used and/or analyzed during the current study are available from the corresponding author on reasonable request.

## Abbreviations

DRF: Distal radius fracture
ED: Emergency Department
PIF: Patient Information Folder
ISS: Injury Severity Score
PA: Posterior-anterior
PACS: Picture Archiving and Communication System
ICC: Intraclass Correlation Coefficient
NRS: Numeric Rating Scale
Q-DASH: Quick Disabilities of the Arm, Shoulder and Hand
PRWHE: Patients-Rated Wrist Hand Evaluation
EQ-5D-5L: 5-level EuroQol
iMCQ: Medical Consumption Questionnaire
iPCQ: Production Consumption Questionnaire
ROM: Range of motion
DRUJ: Distal radioulnar joint
SAE: Serious adverse event
CCMO: Central Committee on Research Involving Human Research
CRPS: Complex regional pain syndrome
CEA: Cost-effectiveness analysis CUA Cost-utility analysis
ICER: Incremental cost-effectiveness ratio
QALY: Quality-adjusted life year
